# The Difference in the Changes of Indoxyl Sulfate after Catheter Ablation among Atrial Fibrillation Patients with and without Kidney Dysfunction

**DOI:** 10.1038/s41598-020-57421-z

**Published:** 2020-01-16

**Authors:** Hideki Koike, Toshisuke Morita, Junko Tatebe, Ippei Watanabe, Masaya Shinohara, Toshio Kinoshita, Hitomi Yuzawa, Takeya Suzuki, Tadashi Fujino, Takanori Ikeda

**Affiliations:** 1 Department of Cardiovascular Medicine, Toho University Faculty of Medicine, Tokyo, Japan; 2 Department of Laboratory Medicine, Toho University Faculty of Medicine, Tokyo, Japan

**Keywords:** Interventional cardiology, Atrial fibrillation, Kidney diseases

## Abstract

Indoxyl sulfate (IS), a protein-bound uremic toxin, induces chronic kidney disease (CKD) and atrial fibrillation (AF). Catheter ablation (CA) of AF improves the renal function. However, the transition of uremic toxins is unclear. This study aimed to investigate the transition of the serum IS level in AF patients with and without CKD after CA. A total of 138 consecutive AF patients who underwent CA and maintained sinus rhythm were prospectively enrolled (paroxysmal AF 67.4%). The patients were divided into 4 groups (non-CKD/low-IS:68, non-CKD/high-IS:28, CKD/low-IS:13, and CKD/high-IS:29). The plasma IS levels and estimated glomerular filtration rate (eGFR) were determined before and 1-year after CA. CKD was defined as CKD stage III and a high-IS according to the mean IS (IS ≥ 1.1 μg/ml). CA significantly improved the eGFR in CKD patients (p < 0.001). The serum IS level in the non-CKD/high-IS group was significantly decreased (from 1.7 ± 0.7 to 1.1 ± 0.6 μg/ml, p < 0.001). However, the serum IS level in the CKD/high-IS group did not improve (from 1.9 ± 0.9 to 1.7 ± 0.7 μg/ml, p = 0.22). The change in the IS in the CKD patients significantly differed from that in those without CKD. In the CKD patients, CA did not significantly decrease the IS, a risk factor of CKD, regardless of an improved eGFR.

## Introduction

Indoxyl sulfate (IS) is a uremic toxin and bounds predominantly to albumin. Furthermore, IS is not a well dialyzable substance^1^. On the other hand, dietary tryptophan is metabolized into IS in our body and it is also present in healthy persons^1–6^. IS is excreted into the urine in the healthy kidney, therefore, in patients with chronic kidney disease (CKD), especially with a renal tubular excretory dysfunction, IS easily accumulates in the body^7^. Accumulation of IS has been proposed to accelerate the fibrosis in various tissues, therefore, induces not only the progression of CKD but also cardiovascular disease and atrial fibrillation (AF)^2,3,8–12^.

There are many reports that renal dysfunction is a critical factor for developing AF^13–16^. Further radiofrequency catheter ablation (CA) improves the renal function in patients with AF^13–18^. However, the precise mechanism of improving renal function, such as a transition of uremic toxins is unclear. We previously reported the relationship between IS and renal function in patients without CKD^19^. On the other hand, the transition of the serum IS level in patients with CKD has not been fully elucidated. The purpose of this present study is to investigate the difference in the transition of the renal function and serum IS level in AF patients with/without CKD after CA.

## Methods

### Study population and study design

Of a total of 183 consecutive AF patients who underwent CA at our institute (Toho University Medical Center Omori Hospital, Tokyo, Japan) between January 2016 and December 2017, 45 who had recurrent AF during the follow-up period were excluded. Finally, 138 patients who successfully underwent CA and maintained sinus rhythm (SR) for at least one year after the CA were enrolled in this analysis.

The plasma IS levels and estimated glomerular filtration rate (eGFR) were measured before (baseline) and at one year after a successful CA. CKD was defined as CKD stage III (eGFR 30–60 ml/min/1.73 m^2^) and a high-IS was defined according to the mean plasma IS level (IS ≥ 1.1 μg/ml) at baseline. The 138 Patients were divided into four groups according to this definition; non-CKD/low-IS (n = 68), non-CKD/high-IS (n = 28), CKD/low-IS (n = 13), and CKD/high-IS (n = 29). We evaluated the relationship between the IS and eGFR and investigated the serial changes in those markers at one year after a successful CA among the four groups.

The study was in compliance with the principles outlined in the Declaration of Helsinki, and all experiments were performed in accordance with relevant guidelines and regulations. The study protocol was approved by the institutional review board of the Toho University Medical Center Omori Hospital. All patients gave their informed consent for the study protocol.

### Analysis of the serum IS

The serum IS concentrations were determined by using high-performance liquid chromatography (HPLC) (GULLIBER; JASCO Corporation, Tokyo Japan). Each serum sample (10 μL) was analyzed by a reversed-phase HPLC (Capcell Pak MF Ph-1 SG80S5 4.6 mm I.D. × 150 mm; SHISEIDO CO., LTD., Tokyo Japan). The mobile phase, 0.1 M KH_2_PO_4_/Tetrahydrofuran (95/5, V/V) (pH 6.5), was delivered at a flow rate of 1.0 mL/min at 37 °C. The serum IS levels were measured by fluorescence detection (excitation, 295 nm; emission, 390 nm).

### CA procedure

Echocardiography was performed in all patients before the CA. The echocardiographic parameters were measured in the standard parasternal long-axis and 4-chamber views. The left ventricular ejection fraction was calculated by the modified Simpson method. All antiarrhythmic drugs (AADs), except for amiodarone, were stopped for at least seven half-lives prior to the procedure and anticoagulant therapy was effectively administrated for more than one month in all patients. We did not perform a routine contrast cardiac CT before the CA. We used propofol and dexmedetomidine to perform the CA under deep sedation. A 7Fr 20-pole 3-site mapping catheter (BeeAT, Japan-Life-Line, Tokyo, Japan) was inserted into the coronary sinus via the right jugular vein. Further, catheters were introduced percutaneously through the femoral vein and a transseptal procedure was performed to access the left atrium (LA). After a transseptal puncture, a 3.5-mm open irrigated tip ablation catheter was used to perform the extensive pulmonary vein isolation (PVI) with the double-lasso technique. CA was guided with the use of a 3D mapping system (CARTO, Biosense-Webster or EnSite NavX, St. Jude Medical). The endpoint of the PVI was the elimination of all PV potentials between the LA and PVs at least 30 min after the successful PVI. Furthermore, the elimination of any dormant PV conduction was confirmed by adenosine triphosphate. Additionally, an incremental isoproterenol infusion was administrated to identify any induction of AF or any non-PV triggers. When frequent atrial premature contractions originating from non-PV sites were revealed, additional ablation was performed to eliminate any non-PV foci.

### Follow-up after CA

After the first procedure, all patients were followed up every 1–3 months in the outpatient clinic for 12 months after the CA. A 12-lead electrocardiogram, 24-hr-Holter electrocardiogram, and assessment of the symptoms were checked every month within 4–6 months after the CA. Freedom from AF was defined as no detectable AF/atrial tachycardias (ATs) on the electrocardiogram modalities performed multiple times after the final procedure. AF recurrence was defined as sustained AF/AT lasting more than 30 seconds after a three-month blanking period according to the guidelines^20^. The discontinuation of AADs was determined in each patient. However, the AADs were continued for at least 3 months following the ablation in patients with persistent AF. The patients who had recurrent AF during the follow-up period were excluded from this study.

### Statistical analysis

All continuous data were expressed as the mean ± standard deviation, medians (quartile: 25–75%), or numbers (%). Comparisons among the groups were analyzed using a univariate analysis (one-way ANOVA, post-hoc test with Tukey, and Fisher’s exact test) and a multivariate analysis using a multiple linear regression model. A p value < 0.05 was considered statistically significant. The statistical analyses were performed using EZR software (Jichi Medical University, Japan), which is a graphical user interface for R (The R Foundation for Statistical Computing, version 2.13.0)^21^.

## Results

### Baseline characteristics

**Figure 1:**
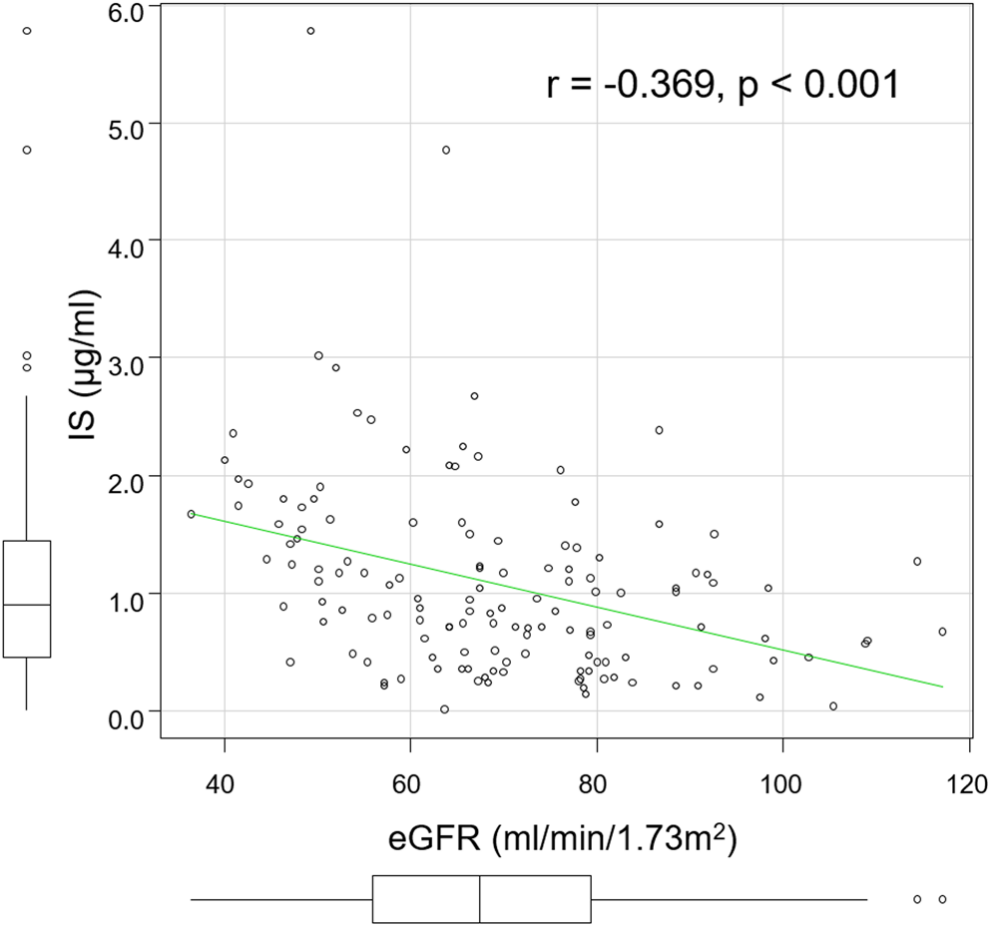
The Correlation between the eGFR and serum IS level. The red colored words show the Pearson product-moment correlation coefficient, r. The asterisks show that the factors had a significant value.

Those baseline characteristics are listed in Table 1. The mean age was 65.5 ± 10.7 years, and 93 (67.4%) were males. Paroxysmal AF was present in 93 patients (67.4%). There was no significant difference in the type of AF among the four groups. The mean eGFR and serum IS levels at baseline were 69.1 ± 16.8 ml/min/1.73 m^2^ and 1.1 ± 0.8 μg/ml, respectively. The serum IS had little correlation with the eGFR in those patients (r = −0.37, P < 0.001, Fig. 1). IS was measured during SR in the 115 patients (83.3%) before CA.

**Table 1:** Baseline Characteristics among the Four Groups.

Factor	non-CKD/low-IS n = 68	non-CKD/high-IS n = 28	CKD/low-IS n = 13	CKD/high-IS n = 29	p. value
Age (years)	62.56 ± 11.46	65.64 ± 10.86	68.15 ± 7.43	71.00 ± 7.19	0.003
Height (cm)	163.49 ± 20.57	166.04 ± 6.45	161.48 ± 9.27	161.95 ± 8.60	0.742
Weight (kg)	63.89 ± 13.14	65.15 ± 8.99	65.92 ± 17.66	60.88 ± 12.66	0.540
Male (%)	48 (70.6)	23 (82.1)	6 (46.2)	16 (55.2)	0.052
PAF (%)	48 (70.6)	19 (67.9)	6 (46.2)	20 (69.0)	0.389
AF duration (Mo)	36.51 ± 39.88	48.64 ± 42.43	36.23 ± 43.77	48.90 ± 62.24	0.502
CHADS_2_ score	0.96 ± 1.07	1.04 ± 0.74	1.15 ± 0.99	1.76 ± 1.18	0.006
HT (%)	27 (39.7)	18 (64.3)	10 (76.9)	18 (62.1)	0.017
CHF (%)	5 (7.4)	2 (7.1)	1 (7.7)	12 (41.4)	<0.001
DL (%)	24 (35.3)	9 (32.1)	7 (53.8)	9 (31.0)	0.512
DM (%)	9 (13.2)	1 (3.6)	0 (0.0)	5 (17.2)	0.192
Stroke (%)	6 (8.8)	1 (3.6)	1 (7.7)	4 (13.8)	0.595
ACE-I/ARB (%)	17 (25.0)	10 (35.7)	5 (38.5)	13 (44.8)	0.252
B blocker (%)	30 (44.1)	9 (32.1)	8 (61.5)	21 (72.4)	0.012
Diuretics (%)	5 (7.4)	2 (7.1)	0 (0.0)	13 (44.8)	<0.001
AADs (%)	50 (73.5)	22 (78.6)	11 (84.6)	22 (75.9)	0.835
Cr (mg/dl)	0.74 ± 0.14	0.77 ± 0.12	0.93 ± 0.15	1.05 ± 0.22	<0.001
eGFR (ml/min/1.73 m^2^)	78.06 ± 13.31	75.58 ± 11.94	53.79 ± 4.17	48.53 ± 5.54	<0.001
IS (μg/ml)	0.6 ± 0.3	1.7 ± 0.7	0.6 ± 0.3	1.9 ± 0.9	<0.001
BNP (pg/dl)	58.51 ± 66.73	82.21 ± 102.87	93.32 ± 92.96	127.76 ± 99.58	0.004
EF (%)	67.53 ± 7.89	68.36 ± 12.29	65.35 ± 11.18	63.87 ± 13.12	0.325
LVDd (mm)	48.47 ± 5.84	51.22 ± 5.37	49.55 ± 3.81	49.49 ± 7.27	0.234
LVDs (mm)	30.06 ± 4.88	31.08 ± 6.03	31.33 ± 4.17	31.72 ± 7.85	0.579
LAD (mm)	36.84 ± 6.96	38.27 ± 6.77	38.82 ± 7.90	41.21 ± 6.66	0.048
Total Procedure time (min)	172.78 ± 33.15	161.04 ± 34.68	157.00 ± 24.79	166.00 ± 32.16	0.267
Contrast agent (ml)	24.43 ± 3.72	45.70 ± 100.98	26.27 ± 4.15	22.61 ± 6.01	0.200
Non-PV triggers (%)	26 (38.2)	4 (14.3)	5 (38.5)	11 (37.9)	0.125
Post_ACE-I/ARB (%)	14 (20.6)	10 (35.7)	5 (38.5)	12 (41.4)	0.135
Post_B blocker (%)	25 (36.8)	7 (25.0)	6 (46.2)	19 (65.5)	0.013
Post_Diuretics (%)	3 (4.4)	2 (7.1)	0 (0.0)	11 (37.9)	<0.001
Post_AADs (%)	15 (22.1)	7 (25.0)	5 (38.5)	7 (24.1)	0.662

PAF, paroxysmal atrial fibrillation; HT, hypertension; CHF, congestive heart failure; DL, dyslipidemia; DM, diabetes mellitus; ACE-I/ARB, angiotensin-converting-enzyme inhibitor/angiotensin receptor blocker; AADs, anti-arrhythmia drugs; eGFR, estimated glomerular filtration rate; IS, indoxyl sulfate; BNP, B-type natriuretic peptide; EF, ejection fraction; LVDd/Ds, left ventricular diameter (diastolic/systolic); LAD, left atrial diameter; PV, pulmonary vein; Data are expressed as the mean ± SD or number (%).

The number of patients with hypertension (HT) in the non-CKD/low-IS group was lower than that in the others. In the CKD/high-IS group, the number of chronic heart failure patients was significantly greater than that in the others. Further, their CHADS_2_ scores were also higher than that in the others. There was a significantly greater number of patients who were administrated beta-blockers and diuretics after the CA in the CKD/high-IS group than the others (Table 1).

### The transition in the eGFR and indoxyl sulfate

We investigated the serial change in the serum IS level and eGFR at one year after the CA. CA significantly improved the eGFR in the patients with CKD (from 50.2 ± 5.7 to 55.4 ± 10.8 ml/min/1.73 m^2^, p < 0.001), as compared to the eGFR in the patients without CKD (from 77.3 ± 12.9 to 75.7 ± 12.7 ml/min/1.73 m^2^, p = 0.11).

**Figure 2:**
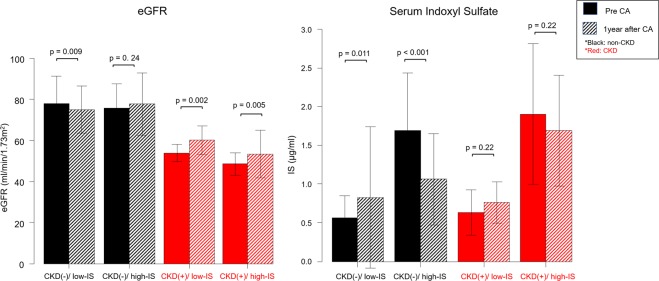
Comparison of the eGFR and serum indoxyl sulfate level between that before and after CA. The black colored words and figure show the patients with non-CKD, and the red colored words and figure show the patients with CKD. The Diagonal lined figures represent one year after the catheter ablation.

**Figure 3:**
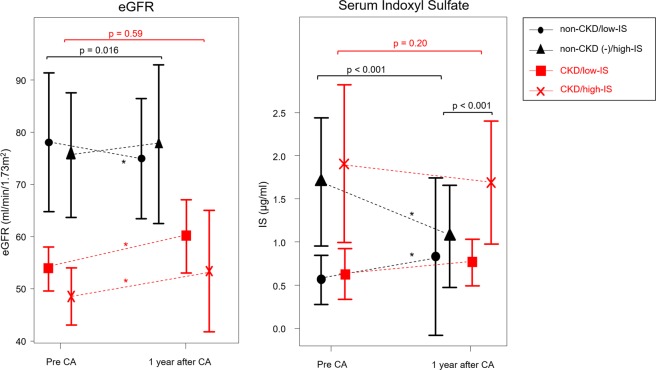
Serial change in the eGFR and serum indoxyl sulfate level. The black colored words and figure show the patients with non-CKD, and the red colored words and figure show the patients with CKD. The circles, triangles, squares, and cross marks represent non-CKD/low-IS, non-CKD/high-IS, CKD/low-IS, and CKD/high-IS, respectively.

Figure 2 shows the comparison of the eGFR and IS before and after the CA among the four groups. Figure 3 also demonstrates the transition in these markers, which was analyzed by a repeated measure ANOVA analysis of variance. In the non-CKD group, the eGFR in the patients with a high-IS level increased after the CA, compared to the eGFR in the patients with a low-IS level (p = 0.016, Fig. 3). On the other hand, in the CKD group, the eGFR significantly improved in both the patients with a high-IS level and those with a low-IS level, however, there was no significant difference in the change rate of the eGFR between the high-IS and low-IS groups with CKD (p = 0.59, Fig. 3).

In the non-CKD group, the serum IS level in the patients with a high-IS significantly improved (from 1.7 ± 0.7 to 1.1 ± 0.6 μg/ml, p < 0.001). However, in the CKD group, the serum IS level in the patients with a high-IS did not significantly improve (from 1.9 ± 0.9 to 1.7 ± 0.7 μg/ml, p = 0.22) regardless of an improved eGFR (Fig. 2). Figure 3 also demonstrates that the serum IS level in the patients with CKD did not change before and after the CA (p = 0.20), but the serum IS level in the patients with non-CKD/high-IS significantly decreased (p < 0.001). Furthermore, the serum IS level in the patients with CKD/high-IS was significantly higher than that in the other three groups (p < 0.001, Fig. 3).

We also evaluated the relationship between the change in the IS and eGFR measured before and after the CA, ΔIS, and ΔeGFR, respectively. The ΔIS was related to the ΔeGFR (r = 0.39, p < 0.001).

The multiple regression analysis after adjusting for the background of the patients, administering drugs after the CA, and the serum IS level revealed that the ΔeGFR had a significant and strong association with the ΔIS (Table 2). Table 3 also shows the multiple regression analysis of the ΔIS after adjusting for the same clinical factors. It demonstrated that the serum IS level at baseline, ΔeGFR, CHF, and post-diuretics had a significant relationship to the ΔIS, but the ΔIS was independent of the eGFR at baseline. At one year after CA, anticoagulants were discontinued in the 60.1% of all patients. However, there was no significant relationship between ΔIS and anticoagulants.

**Table 2:** Multiple Linear Regression Model of the ΔeGFR.

Factor	Univariable analysis		Multivariable analysis	
	Estimate β	p value	Estimate β	p value
ΔIS	43.76*	<0.001	41.06*	<0.001
eGFR	−0.25*	<0.001	−0.22*	<0.001
IS	2.83*	0.004	−1.35	0.246
CHADS_2_ score	0.18	0.822	−0.63	0.558
Age	0.04	0.574	−0.01	0.931
CHF	5.15*	0.029	1.12	0.683
HT	0.60	0.718	0.55	0.765
Post_Diuretics	2.33	0.372	0.98	0.749
Post_β blocker	5.39*	0.001	3.01	0.053

HT, hypertension; CHF, congestive heart failure; eGFR, estimated glomerular filtration rate; IS, indoxyl sulfate; *had a significant value by the multiple linear regression model.

**Table 3:** Multiple Linear Regression Model of the ΔIS.

Factor	Univariable analysis		Multivariable analysis	
	Estimate β	p value	Estimate β	p value
ΔeGFR	0.004*	<0.001	0.003*	<0.001
IS	0.054*	<0.001	0.059*	<0.001
eGFR	−0.001	0.067	0.0003	0.456
CHADS_2_ score	−0.011	0.104	−0.340	0.735
Age	−0.001	0.139	−0.001	0.653
CHF	0.031	0.147	0.034	0.113
HT	−0.012	0.438	−0.006	0.674
Post_Diuretics	−0.045	0.052	−0.103*	<0.001
Post_β blocker	0.018	0.226	0.012	0.313

HT, hypertension; CHF, congestive heart failure; eGFR, estimated glomerular filtration rate; IS, indoxyl sulfate; *had a significant value by the multiple linear regression model.

## Discussion

The main findings were as follows. First, in the patients with CKD, CA improved their eGFR as in the previous studies. Second, the change in the IS in the patients with CKD significantly differed from that in those without CKD. In the non-CKD group, the IS level improved after the CA. On the other hand, the serum level of the IS in the CKD group did not improve regardless of improving their eGFR, even though IS is generally excreted from the kidneys. In patients with CKD, IS, which is a protein-bound uremic toxin and facilitates renal dysfunction, remained after the CA. These findings indicated that renal dysfunction in patients with CKD may not be improved by CA, and CKD may proceed after CA.

CKD increases the incidence and prevalence of AF. Furthermore, AF causes a decrease in the eGFR^13–16^. Hypertension and diabetes are known as risk factors of subsequent AF and CKD, and both associations had a significant value in the patients without hypertension or diabetes^16^. In this present study, there were no significant differences in the prevalence of diabetes among the four groups, however the prevalence of hypertension was significantly greater in the patients with CKD or a high-IS. However, a multiple regression analysis revealed that hypertension was not a significant factor associated with the ΔeGFR (Table 2).

Some studies reported other mechanisms of AF development in CKD patients. Those were proposed to be inflammation and oxidant stress due to IS. IS, a highly protein-bound uremic toxin, induces activation of NADPH oxidases and the production of reactive oxygen species, and as a result, IS directly exacerbates the oxidative stress^22,23^. Therefore, IS increases cardiac fibrosis and the development of AF substrates^3,24^.

IS is excreted from the circulation into the urine in healthy kidneys^7^. IS generally is metabolized by dietary tryptophan^12^. Therefore, a high-protein diet and gut-microflora affect and increase the serum IS level in patients with mild renal dysfunction or without CKD^5,6^. However, in patients with renal dysfunction, especially with an impaired renal tubular excretory function, IS easily accumulates in their bodies^7^. Patients with advanced CKD have more increased serum IS levels than patients without CKD. Further, the accumulated IS increases and is associated with a future risk^3,25^. Therefore, CKD causes the development of AF due to an AF substrate caused by IS toxicity^3^.

In this present study, there were a larger number of patients with chronic heart failure among those with CKD/high-IS, and their left atrial diameter (LAD) was larger. Further, the number of patients with hypertension was significantly greater in those with CKD or a high-IS. That suggested that in the real world, IS exacerbates the AF substrate or fibrosis.

CKD and AF share risk factors and have common pathophysiologic processes that induce both outcomes. CKD increases the prevalence of AF, and AF increases the risk of the development of CKD. The mechanisms of the associations between CKD and AF are well known^13–16^. Further, it has been well reported that successful CA of AF improves the renal function in patients with CKD^15,17^. Some reports have demonstrated that the improvement in the LA function and cardiac output contribute to the improvement in the renal function. In AF patients with CKD, the left atrial (LA) contractile function and LA appendage velocity are reduced, and those patients present with a higher prevalence of a spontaneous contrast echo^26^. CA improved the left atrial function or cardiac output, as a result, the effect of CA can bring about the improvement in the renal function^27–29^. However, other or precise mechanisms have remained unclear.

Our study demonstrated that the eGFR in patients with non-CKD/high IS improved and their serum IS level also decreased. That suggested that the maintenance of SR after CA might increase the renal blood flow and the IS was washed out and reduced, however, we considered that a reduction in the IS itself prevented the progression of CKD. The reduction in the IS may not be just the result of an improvement in the eGFR, because in the non-CKD patients with a high-IS, the serum IS level decreased and their eGFR significantly improved, as compared to that in those with a low-IS (Fig. 3). Furthermore, the ΔeGFR between that before and after the CA had a strong association with the ΔIS level (Table 2). Those findings supported another mechanism of the improvement in the IS independent of the eGFR. Some reports have pointed out the relationship between the gut-micro flora and IS^30,31^. The worsened hemodynamics impair the gut barrier and cause the intestinal overgrowth of pathogenic bacteria^30,31^. However, there have been no reports about the relationship between the gut-micro flora and ablation or the improvement in the hemodynamics and gut-flora. Our study insisted that an improvement in the hemodynamics after CA might affect the gut-micro flora and contribute to the reduction in the IS.

On the other hand, the serum IS level in patients with CKD did not improve, regardless of the improvement in their eGFR. That suggested that in patients with CKD, their actual renal function might not have improved, or an impaired renal tubular excretory function might have remained, because IS is generally excreted from the renal tubules, and the serum IS level in patients with CKD still remains high after CA. On the other hand, the total serum IS level did not change among the CKD and non-CKD groups. The CKD/low-IS patients showed the same results in the eGFR and IS as the CKD/high-IS patients. And the result of Table 2 showed that baseline IS was not associated with ΔeGFR. It may be pointed out that IS could not be a predictor for the renal function. However, we suppose that this is a very important finding by comparing to the transition of IS and eGFR in the non-CKD patients. Because, when IS was divided into high/low group, there was a significant difference in the transition of IS between the CKD and non-CKD patients. In the non-CKD patients with a high-IS, the serum IS level decreased and their eGFR improved, as compared to that in those with a low-IS (Fig. 3). Those findings may have demonstrated that the renal dysfunction in the CKD/high-IS patients may be irreversible, but the high-IS level in the non-CKD patients may be treatable and could improve with CA. Thus, in patients with CKD, their actual renal function might not have improved and the renal function in the CKD/high-IS patients might decrease again in the further future more than that in the CKD/low-IS patients. Because IS, which induces the progression of CKD, remained high after CA in patients with CKD. Although there may be a limitation of short follow-up term. Because we analyzed those patients for only one year, therefore we need more long-term follow-up. However, those findings suggested that IS may be a useful marker for evaluating the renal function in CKD patients, independent of the eGFR. Thus, it is reasonable that Table 2 revealed that ΔIS was associated with ΔeGFR, although baseline IS was not associated with ΔeGFR. And the results of a multiple analysis (Table 3) for the ΔIS also supported this suggestion, because the ΔIS was independent of the baseline eGFR. Furthermore, it suggests that high IS level in the non-CKD patients before CA could be a significant predictor of the preventing progression of CKD due to a reduction in the IS level after CA.

### Study limitations

This study had some potential limitations. First, this study was a single center trial. We did not have very many patients. Second, the precise mechanism of the improvement in the renal function remained unclear. IS directly exacerbates the oxidative stress, however, the impact of IS on the fibrosis or oxidative stress remains unclear in the clinical cases. Because in our study, we did not evaluate the markers of oxidative stress, such as NO or endothelium-derived relaxing factors (EDRFs). Furthermore, we did not measure the cardiac output or renal flow before and after the CA in this study. Therefore, we could not completely deny that the reduction in the IS was just the result by the improvement in the eGFR. Additionally, we could not evaluate the markers of renal tubular dysfunction. However, the transition in the IS and eGFR in the patients with and without CKD suggested that the mechanism of the improvement in the renal function was as follows. The maintenance of SR after CA might have increased the renal blood flow and the IS was washed out and reduced, and a reduction in the IS itself prevented the progression of CKD, especially in patients without CKD. Further research is needed with a greater number of patients to confirm our findings.

## Conclusions

The change in the serum IS level in the patients with CKD significantly differed from that in those without CKD. In the patients with CKD, CA improved their eGFR, however, the serum level of IS, which is a protein-bound uremic toxin and is excreted from the renal tubules, did not improve after CA. These findings suggested that the actual renal dysfunction in the CKD/high-IS patients may be irreversible, however, CA improved the eGFR. Furthermore, IS may be a useful marker for evaluating the renal function, independent of the eGFR.

## Data Availability

The datasets generated and/or analyzed during the current study are available from the corresponding author upon reasonable request.
